# A novel pH‐controlled hydrogen sulfide donor protects gastric mucosa from aspirin‐induced injury

**DOI:** 10.1111/jcmm.13166

**Published:** 2017-04-07

**Authors:** Chun‐tao Yang, Zhen‐zhen Lai, Ze‐hang Zheng, Jian‐ming Kang, Ming Xian, Rui‐yu Wang, Kun Shi, Fu‐hui Meng, Xiang Li, Li Chen, Hui Zhang

**Affiliations:** ^1^ Affiliated Cancer Hospital & Institute Guangzhou Medical University Guangzhou China; ^2^ Key Laboratory of Protein Modification and Degradation School of Basic Medical Sciences Guangzhou Medical University Guangzhou China; ^3^ NanShan School of the First Clinical College Guangzhou Medical University Guangzhou 510120 China; ^4^ Department of Chemistry Washington State University Pullman WA USA; ^5^ Quality Control Section of Academic Affairs Guangzhou Medical University Guangzhou China

**Keywords:** gastric lesions, inflammation, mucosal defence, NSAIDs, oxidative stress, pH‐controlled H_2_S donors

## Abstract

Hydrogen sulphide (H_2_S) serves as a vital gastric mucosal defence under acid condition. Non‐steroidal anti‐inflammatory drugs (NSAIDs) are among widely prescribed medications with effects of antipyresis, analgesia and anti‐inflammation. However, their inappropriate use causes gastric lesions and endogenous H_2_S deficiency. In this work, we reported the roles of a novel pH‐controlled H_2_S donor (JK‐1) in NSAID‐related gastric lesions. We found that JK‐1 could release H_2_S under mild acidic pH and increase solution pH value. Intragastrical administration of aspirin (ASP), one of NSAIDs, to mice elicited significant gastric lesions, evidenced by mucosal festering and bleeding. It also led to infiltration of inflammatory cells and resultant releases of IL‐6 and TNF‐α, as well as oxidative injury including myeloperoxidase (MPO) induction and GSH depletion. In addition, the ASP administration statistically inhibited H_2_S generation in gastric mucosa, while up‐regulated cyclooxygenase (COX)‐2 and cystathionine gamma lyase (CSE) expression. Importantly, these adverse effects of ASP were prevented by the intragastrical pre‐administration of JK‐1. However, JK‐1 alone did not markedly alter the property of mouse stomachs. Furthermore, *in vitro* cellular experiments showed the exposure of gastric mucosal epithelial (GES‐1) cells to HClO, imitating MPO‐driven oxidative injury, decreased cell viability, increased apoptotic rate and damaged mitochondrial membrane potential, which were reversed by pre‐treatment with JK‐1. In conclusion, JK‐1 was proved to be an acid‐sensitive H_2_S donor and could attenuate ASP‐related gastric lesions through reconstruction of endogenous gastric defence. This work indicates the possible treatment of adverse effects of NSAIDs with pH‐controlled H_2_S donors in the future.

## Introduction

In general, stomachs can resist a variety of unimaginable lesions induced by hydrochloric acid (HCl), refluxed bile salts, alcohol etc. This is attributed to a very important barrier, mucus‐bicarbonate barrier. Once the barrier is damaged by certain unusual stimuli, a cascade of severe gastric mucosal lesions will take place, including activation of pepsinogen, digestion even erosions or ulcers of mucosa, as well as inflammatory and oxidative injury 1.

Among those unusual stimuli leading to gastric mucosal lesions, NSAIDs are especially important. As we know, NSAIDs have high efficacy in antipyresis, analgesia, anti‐inflammation and antirheumatism. In addition, studies have indicated that NSAIDs have promising activities for coagulation, stroke and myocardial ischaemia 2. Therefore, they have widely been prescribed in clinic throughout the world. However, their inappropriate use (*e.g*. overdose, long‐term or wrong formulations) is a major reason for stomach lesions, evidenced by gastric mucosal erosion, haemorrhage, even ulcers 3, 4. Gastric lesions induced by NSAIDs are associated with inhibition of endogenous COX, prostaglandin (PG) and H_2_S synthesis, as well as infiltration of inflammatory cells and oxidative injury 5, 6, 7, 8, 9.

H_2_S, much like nitric oxide (NO), has been recognized as a critical gaseous signalling molecule. It plays regulatory roles in a stomach and many other organs, for instance, relaxation of blood vessels, antioxidation and regulation of inflammation 10, 11. Consequently, to overcome NSAIDs’ detrimental effects, researchers have developed many NSAID‐hybrid drugs that can release H_2_S 5, 12, NO 13, 14 or both 15, 16. Those hybrids have showed improved pharmacological activities and fewer side‐effects. It should be noted, however, this strategy is not always simple. In addition, inappropriate modifications of drugs may be at risk of reduction in therapeutic effects. Most of these drugs are oral medicine and broken down in intestinal tracts. We surmised that if a new designed compound is disintegrated in stomach, it will provide a high local H_2_S level and easily exert gastric protection.

In view of the aforementioned problem, we feel it is still reasonable and simple to just apply H_2_S when NSAIDs are orally administered. For instance, a H_2_S donor, NaHS, protects oesophagus and oesophagogastric junction against naproxen‐induced injury 9. However, as traditional H_2_S donors, NaHS or other sulphide salts can be easily oxidized to form sulfane sulphurs. Moreover, these salts are fast donors which release H_2_S immediately upon dissolving in solutions. As H_2_S is volatile, it is hard to obtain a precise H_2_S concentration. As for another commonly used H_2_S donor GYY4137, it releases H_2_S upon spontaneous hydrolysis and this process is slow and uncontrollable 17. To achieve a better control of H_2_S release and in the light of the acidic environment in stomachs, we recently developed a series of pH‐controlled H_2_S donors. We found increased acidic conditions could significantly promote H_2_S generation from these donors and some of them have been shown promising activity against myocardial ischaemia‐reperfusion injury 18.

Encouraged by these studies, we turned to explore the applications of these donors in gastric protection, given the fact that stomach maintains an acidic environment with pH < 2. In this study, the representative pH‐controlled H_2_S donor JK‐1 was first prepared and characterized. Then, its H_2_S release profile under different pH values and impact on solution pH value were measured. Next, a model of acute gastric mucosal lesions was established by administered intragastrically aspirin (ASP) to mice and gastric protective effects of JK‐1 were investigated. Finally, JK‐1‐mediated gastric protection was further evaluated in an *in vitro* model, incubation of gastric mucosal epithelial (GES‐1) cells with hypochloric acid (HClO), to imitate MPO‐driven oxidative injury. Both *in vivo* and *in vitro* studies showed that JK‐1 attenuated ASP‐related gastric lesions through reconstruction of endogenous gastric defence.

## Materials and methods

### Materials

ASP, Hoechst 33258 and sodium hypochlorite (HClO) solution were purchased from Sigma‐Aldrich Co. (St. Louis, MO, USA). Commercial enzyme‐linked immunosorbent assay (ELISA) kits were supplied by Boster BioTech Co. (Wuhan, China) for measuring MPO, interleukin (IL)‐6 and tumour necrosis factor (TNF)‐α. Primary antibody against COX‐2 or CSE was purchased from Bioworld Technology Inc. (Louis Park, MN, USA). β‐Actin was used as a loading control, whose primary antibody was purchased from KangChen Bio‐tech Inc. (Shanghai, China). Cell counting kit (CCK)‐8 and rhodamine (Rh)123 were purchased from Dojindo Laboratory (Kyushu, Japan). DMEM and foetal bovine serum (FBS) were supplied by Gibico BRL (Ground Island, NY, USA). Other compounds were provided by Thermo Fisher Scientific Inc. (Shanghai, China).

### Synthesis of JK‐1

**Figure 1:**
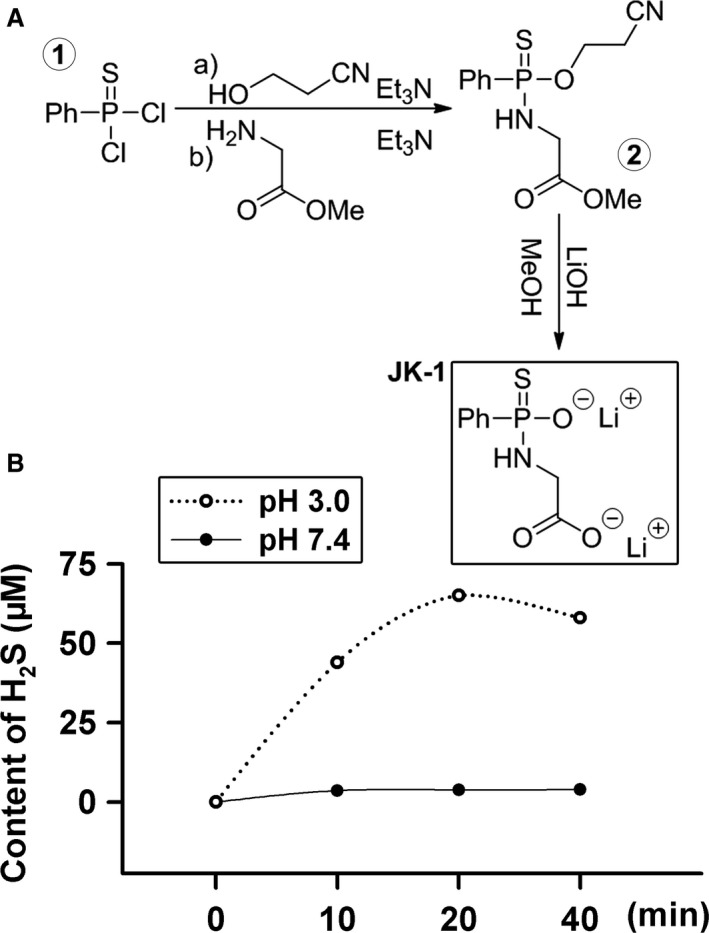
Synthesis and release of a pH‐controlled hydrogen sulphide donor JK‐1. (**A**) Synthesis of JK‐1. (**B**) Effects of pH on H_2_S release from JK‐1. JK‐1 solutions (100 μM) were prepared and confected to pH 7.4 and pH 3.0, respectively. The released H_2_S, at 0, 10, 20 and 40 min., from the solutions, was measured by the methylene blue assay.

JK‐1 was prepared from phosphonothioic dichloride in two steps (Fig. 1A), following the reported protocols 18 with some modifications to increase the yield:

Preparation of the intermediate ②: Phosphonothioic dichloride (0.45 ml, 3 mmol) was dissolved in 3.5 ml of anhydrous dichloromethane (CH_2_Cl_2_). To this solution was added 3‐hydroxypropionitrile (0.2 ml, 3 mmol) and triethylamine (0.45 ml, 3 mmol) under an Ar_(g)_ atmosphere at 0°C . The reaction was stirred at 0°C for 15 min. and at room temperature for 4 hrs. A solution of glycine methyl ester (0.414 g, 3.3 mmol), triethylamine (1.25 ml, 9 mmol) and 3.5 ml anhydrous dichloromethane (CH_2_Cl_2_) was added, and the reaction was stirred for an additional 2 hrs. The mixture was diluted with 10 ml dichloromethane (CH_2_Cl_2_), washed with 10 ml of 2 M H_2_SO_4_, dried (MgSO_4_) and concentrated under reduced pressure. The crude material was purified by flash chromatography using 0.5% ethyl acetate in dichloromethane to afford the intermediate ② as a yellow oil (608 mg, 68%), whose NMR data were the same as reported 18. The intermediate ② (150 mg, 0.5 mmol) was dissolved in 2 ml of methanol. To this mixture was added 2 ml of 1 M LiOH aqueous solution (freshly prepared). The resultant solution was stirred at room temperature for 12 hrs and concentrated to dryness. The resulting solid was suspended in anhydrous methanol, filtered (0.2 μm Teflon membrane) and concentrated. The final product JK‐1 was obtained as white solid in 90% yield after lyophilization. Its NMR data were the same as reported 18.

### Measurement of acid‐stimulated H_2_S release from JK‐1

H_2_S generation from JK‐1 was initiated by adding 50 μl of freshly prepared donor stock solution (30 mM in DI water) into 15 ml of DI water (pH 3.0 and 7.4). Then, 1.0 ml of the solution aliquots was periodically taken and transferred to 1.5‐ml EPPENDORF tubes containing 100 μl of 1% zinc acetate and 5 μl of NaOH solution (5 M). This was followed by centrifugation at 20,500 rcf for 20 min. to pellet the zinc sulphide that was formed. The supernatant was then removed and the pellet reconstituted with 200 μl of *N, N*‐dimethyl‐1,4‐phenylenediamine sulphate (20 mM in 7.2 M HCl) and 200 μl of ferric chloride (30 mM in 1.2 M HCl). The methylene blue reaction was carried out for 15 min., and then, the optical density value at 670 nm was determined. H_2_S concentration of each sample was calculated against a calibration curve obtained by a series of Na_2_S solutions.

As we know, gastric juice is an acidic environment necessary for various functions of stomach. Therefore, we test the release H_2_S from JK‐1 under acidic conditions. As shown in Figure 1B, at a mild acidic pH (3.0), JK‐1 released significant amounts of H_2_S. In contrast, JK‐1 produced barely detectable H_2_S under physiological pH (7.4) within the same time.

### Experimental animals

Healthy adult male Kunming mice, weighting 20.0–25.0 g, were obtained from Guangzhou University of Chinese Medicine (Guangzhou, China). They were kept in a SPF level room, which was controlled temperature (22 ± 2°C), humidity (65–70%) and light/dark cycle (12 hrs/12 hrs). All the experiment operations were approved by the Animal Care committee of Guangzhou Medical University.

### Drug treatments and experimental design

The mice were acclimated to the new laboratory environment for at least 2 weeks, when they had free access to food and drinking water. Before the experiments, the mice were fasted for 36 hrs with free access to water. And then, they were randomly divided into four treatment groups: control group, ASP group, JK‐1 + ASP group and JK‐1 alone group.

ASP and JK‐1 were dissolved in 0.5% carboxymethyl cellulose (CC) and phosphate‐buffered solution (PBS), respectively. In *control group*, the mice were administered intragastrically with PBS, and 1 hr later, they were then administered intragastrically 0.5% CC; in *ASP group*: the mice were administered intragastrically with PBS, and 1 hr later, they were then administered intragastrically with 200 mg/kg ASP; In *JK‐1 + ASP group*: the mice were administered intragastrically with 150 μg/kg JK‐1, and 1 hr later, they were then administered intragastrically with 200 mg/kg ASP. We chose the dose of 150 μg/kg JK‐1 based on our previous study 18 and our preliminary experiment. In our previous study, we used 50 and 100 μg/kg JK‐1 in *in vivo* experiments and 12~50 μM in *in vitro* experiments. When we performed *in vivo* preliminary experiment, we used three concentrations of JK‐1, that is 50, 100 and 150 μg/kg, and found 150 μg/kg of JK‐1 has maximal and stable effects; in *JK‐1 alone group*: the mice were administered intragastrically with 150 μg/kg JK‐1, and 1 hr later, they were then administered intragastrically with 0.5% CC.

### Observation of acute gastric mucosa damage

After the above treatments, the mice were killed by injecting intraperitoneally an overdose of pentobarbital sodium (60 mg/kg). The stomachs were removed and cut open along greater curvature. The images of all the samples were captured randomly, and the area of gastric haemorrhagic lesions was counted with Image J software. The infiltration of inflammatory cells and the morphology of damaged gastric tissues were observed through H&E‐stained sections. These assessments were all performed in a blind manner.

### ELISA for IL‐6, TNF‐α and MPO

At the end of treatments, the mice were killed by injecting intraperitoneally an overdose of pentobarbital sodium. The stomachs were opened, and the haemorrhagic gastric mucosa was collected and split. The lysate was used to measure the content of IL‐6, TNF‐α and MPO with commercial ELISA kits according to the manufacturers’ instructions. The optical density values were detected with a microplate reader (Molecular Devices, Sunnyvale, CA, USA). The amounts of the above special proteins were further normalized with total protein levels.

### Measurement of H_2_S generation in gastric mucosa

After administered intragastrically with ASP in the absence or presence of the intragastrical pre‐administration with JK‐1, the mice were killed by injecting an overdose of pentobarbital sodium and their gastric mucosa was collected and split. The lysate was used to measure H_2_S generation as described in our previous paper 19. After incubation of the lysate with 10 mM substrate L‐cysteine and 2 mM coenzyme pyridoxal 5′‐phosphate for 2 hrs in reaction bottles, 400 μl of zinc acetate solution trapped H_2_S was collected and 40 μl of *N, N*‐dimethyl‐1,4‐phenylenediamine sulphate (20 mM in 7.2 M HCl) was added, immediately followed by 40 μl of ferric chloride (30 mM in 1.2 M HCl). The methylene blue reaction was carried out for 15 min., and the optical density value of 670 nm was measured with a microplate reader (Molecular Devices). The generated H_2_S was normalized according to the protein concentration of indicated groups.

### Western blot assay for COX‐2 and CSE expression

At the end of indicated treatments, the mouse gastric mucosa was collected and split. Total proteins in the cell lysate were quantitated with a commercial BCA kit. The loaded proteins were fractionated with 12% sodium dodecyl sulphate‐polyacrylamide gel electrophoresis and then transferred into polyvinylidene difluoride membranes. The membranes were blocked with 5% fat‐free milk (in TBS‐T) for 2 hrs at room temperature and then incubated with the primary antibody against COX‐2 (1:2000) or CSE (1:1000) with gentle agitation overnight at 4°C. Following three washes with TBS‐T, the membranes were incubated with HRP‐conjugated secondary antibodies (1:5000) for 1 hr at room temperature. The immunoreactive signals were visualized with an enhanced chemiluminescence detection system.

### Cell culture

GES‐1 cells are derived from transformation of human gastric mucosal epithelium, which were bought from FuHeng Cell Center (Shanghai, China). The cells were maintained in DMEM supplemented with 10% FBS at 37°C under an atmosphere of 5% CO_2_ and 95% air. They were passaged and harvested with trypsin every other day. In this study, the 3rd to 6th passage number cells were used.

### Determination of cell viability

The cell viability of GES‐1 cells was determined with CCK‐8 assay. Briefly, the cells were plated in 96‐well plates at a density of 10, 000 cells in each well. When they were grown to approximately 70% confluence, the indicated treatments were applied. The CCK‐8 solution (100 μl) at a 1:10 dilution with FBS‐free cell medium was added, and the cells were incubated for 3 hrs at 37°C. Absorbance (*A*) was measured at 450 nm with a microplate reader (Molecular Devices). The mean *A* value of each group was used to calculate percentage of cell viability as follows: Cell viability (%) = (*A*_Treatment group_‐ *A*_Blank_) / (*A*_Control group_‐ *A*_Blank_) ×100. Experiments were performed for at least five times.

### Assessment of cellular apoptosis

Nuclear morphological changes in apoptotic GES‐1 cells, including chromosomal condensation and fragmentation, were observed by Hoechst 33258 staining followed by photofluorography. In brief, at the end of indicated treatments, the cells were fixed with 4% paraformaldehyde in PBS for 10 min. After a careful wash with PBS, the cells were stained with 5 mg/l Hoechst 33258 for 20 min., and then the blue nuclei were visualized under a fluorescent microscope (Advanced Microscopy Group, USA). The nuclei in control cells displayed normal size and uniform fluorescence, whereas apoptotic cell nuclei became condensed, fractured or distorted. Apoptosis rate (%) was calculated by the ratio of the apoptotic cell number to total cell number.

### Measurement of mitochondrial function

Mitochondrial function can be reflected by mitochondrial membrane potential (MMP), which was measured by Rh123 staining followed by photofluorography in this study. Rh123 is a cell‐permeable cationic fluorescent dye entering a mitochondrion based on its highly negative membrane potential. Depolarization of MMP usually reduces Rh123′s intake by mitochondria and generates a relatively weak fluorescence signal. After the treatments of GES‐1 cells, 10 mg/l Rh123 dissolved in FBS‐free medium was added and incubated for 20 min. at 37°C. The generated fluorescent signal was visualized under a fluorescent microscope (AMG, Bothell, WA, USA). The mean fluorescence intensity (MFI) of Rh123 from five random fields in each group was analysed with Image J software (National Institutes of Health, Bethesda, Maryland, USA).

### Statistics

All the data were expressed as mean ± S.D. The statistical significance of intergroup differences was analysed by one‐way analysis of variance (anova) followed by Student–Newman–Keuls test with spss 13.0 software (Chicago, IL, USA). A probability of less than 0.05 was considered statistically significant.

## Results

### JK‐1 increased solution pH through H_2_S release

**Figure 2:**
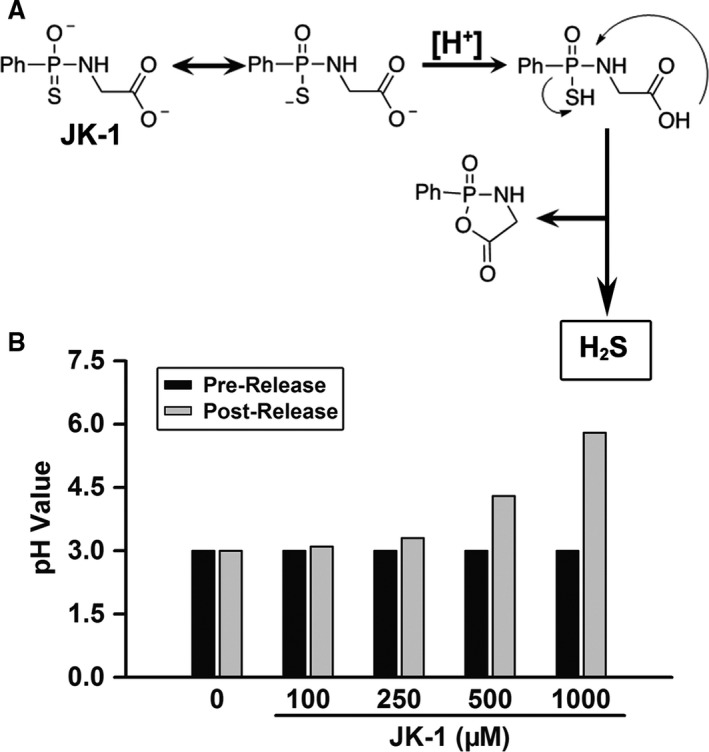
Effects of JK‐1′s H_2_S release on solution pH. (**A**) Proposed acid (H^+^)‐induced H_2_S release mechanism from JK‐1 18. (**B**) In a test tube, 11.8 ml of pH 3.0 aqueous solution was added, followed by the addition of a requisite volume of JK‐1 stock solution to prepare the indicated JK‐1 solutions. The final volume of the reaction solution was adjusted to 12 ml with pH 3 aqueous solution. After mixing and then standing for 60 min. at room temperature, the pH value of each solution was tested.

Based on the proposed reaction mechanism shown in Figure 2A, H_2_S release from JK‐1 could increase the solution's pH due to the consumption of H^+^ ion. This might be protective for gastric mucosa. To prove this hypothesis, we added JK‐1 to pH 3.0 aqueous solutions and their pH values before and after H_2_S release were measured. As shown in Figure 2B, 500 and 1000 μM of JK‐1 significantly increased the solutions’ pH.

### JK‐1 prevented ASP‐induced gastric mucosal injury in mice

**Figure 3:**
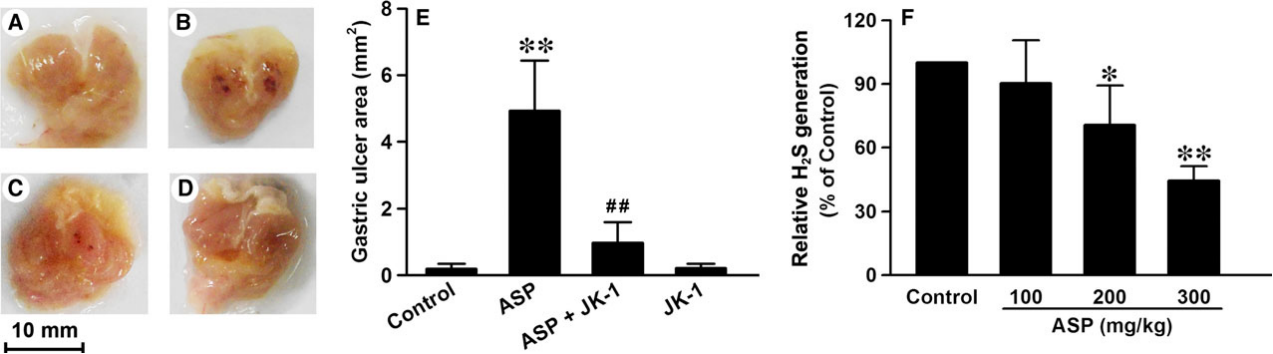
Effects of JK‐1 on ASP‐induced gastric mucosal injury. The mice were fasted for 36 hrs with free access to drinking water and then were treated as *DRUG TREATMENTS AND EXPERIMENTAL DESIGN*. (**A‐D**) The representative images of open stomach samples from the following groups: (**A**) control group, (**B**) ASP group, (**C**) ASP+JK‐1 group, (**D**) JK‐1 group. (**E**) Gastric ulcer area of the four groups was calculated with ImageJ software. (**F**) Mice were administered intragastrically with increasing doses of ASP. The gastric mucosa was collected and split for the measurement of relative H_2_S generation. Data were expressed as mean ± S.D. *n* = 4–6. *P <* *0.05, **P <* *0.01 *versus* control group, ##*P *<* *0.01 *versus* ASP alone group.

As JK‐1 can release H_2_S and also raise pH value, we speculated it might exert a protective effect against NSAIDs‐induced gastric mucosal injury. With this idea in mind, we treated the mice with one of common NSAIDs, ASP, to establish a model of gastric mucosal injury. As shown in Figure 3, intragastric (IG) administration of 200 mg/kg ASP dissolved in 0.5% carboxymethyl cellulose markedly induced gastric mucosal injury, presented as festering and bleeding (B and E). However, the control mice only receiving 0.5% carboxymethyl cellulose did not have any marked gastric mucosal injury (A and E). The data support that the oral administration of ASP can damage gastric mucosal, especially at a large dose. We then wondered whether the endogenous H_2_S defect was involved in the gastric mucosal injury. We therefore measured the ability of gastric mucosal to generate H_2_S. It was found that at low dose (100 mg/kg), the IG administration of ASP did not significantly alter the ability. However, with the dose increasing, ASP‐induced inhibitory effects on the ability to generate H_2_S enhanced gradually (Fig. 3F). Accordingly, we further validated whether JK‐1 had protective effects against ASP‐induced gastric mucosal injury through H_2_S release. The data demonstrated that before the administration of ASP, the treatment of mice with JK‐1 significantly attenuated the gastric mucosal injury, while JK‐1 alone did not cause any obvious injury (Fig. 3C and D).

### JK‐1 alleviated ASP‐induced inflammation response in mouse gastric mucosa

**Figure 4:**
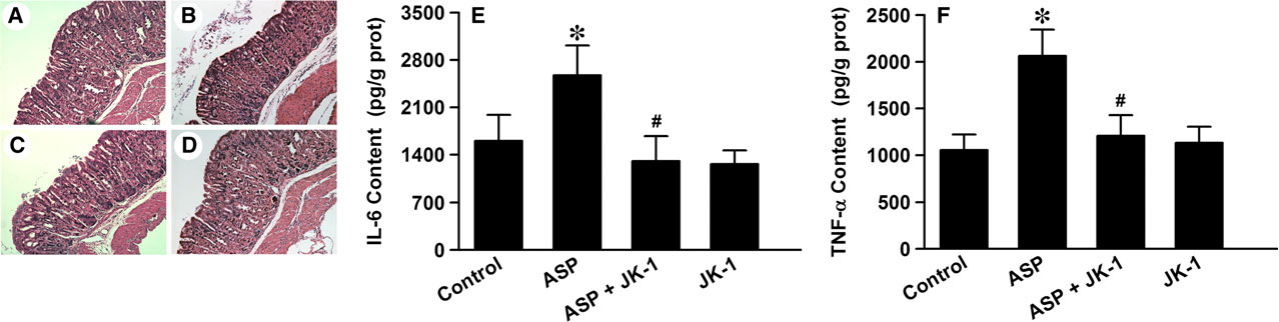
Effects of JK‐1 on ASP‐induced inflammation in gastric mucosa. The mice were fasted for 36 hrs with free access to drinking water and then were treated as *DRUG TREATMENTS AND EXPERIMENTAL DESIGN*. (**A‐D**) The gastric mucosa from the following groups was collected and prepared for H&E staining. (**A**) Control group, (**B**) ASP group, (**C**) ASP+JK‐1 group, (**D**) JK‐1 group. (**E** and **F**) The mouse gastric mucosa from the above groups was cut and split, and the protein samples were extracted for measuring IL‐6 and TNF‐α with the ELISA kits. Data were expressed as mean ± S.D. *n* = 4–6. *P <* *0.01 *versus* control group, #*P *<* *0.01 *versus* ASP alone group.

The micrographs in Figure 4 showed that IG administration of 200 mg/kg ASP markedly damaged the mouse gastric mucosa (B), characterized by the detachment of dead epithelial cells from the normal gastric mucosal tissues, the erosions of mucosal tissues, as well as the infiltration with inflammatory cells, while the gastric mucosa of control mice did not show those manifestations (A). Prior to the administration of ASP, the mice were pre‐administered intragastrically with 150 μg/kg JK‐1. The results showed that the pre‐treatment with JK‐1 significantly mitigated the ASP‐induced injury in gastric mucosa (C), which alone did not trigger any marked injury.

Furthermore, we measured the contents of some pro‐inflammatory factors in the mouse gastric mucosa with ELISA kits. The data showed that the levels of IL‐6 (Fig. 4E) and TNF‐α (Fig. 4F) in gastric mucosa of the mice exposed to ASP were obviously boosted comparing with those in gastric mucosa of the control mice. The elevated levels of IL‐6 (Fig. 4E) and TNF‐α (Fig. 4F) were both able to be attenuated by the pre‐treatment of JK‐1.

### JK‐1 blunted ASP‐induced oxidative stress in mouse gastric mucosa

**Figure 5:**
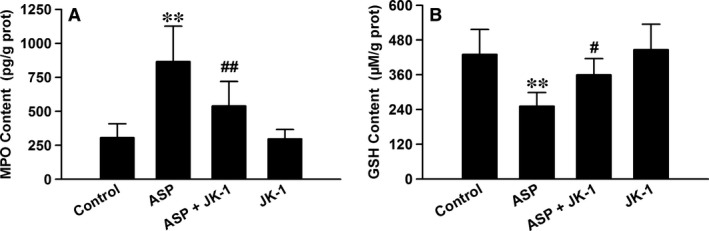
Effects of JK‐1 on ASP‐induced oxidative stress in gastric mucosa. The mice were fasted for 36 hrs with free access to drinking water and then were divided into four groups as *DRUG TREATMENTS AND EXPERIMENTAL DESIGN*: control group, ASP group, ASP+JK‐1 group and JK‐1 group. The mouse gastric mucosa was cut and split, and the protein samples were extracted for testing MPO and GSH contents with the ELISA kits. Data were expressed as mean ± S.D. *n* = 4–6. **P <* *0.01 *versus* control group, #*P *<* *0.05, ##*P *<* *0.01 *versus* ASP alone group.

In damaged gastric mucosal and submucosal layers, inflammatory cells, especially neutrophils and monocytes, are usually accumulated and may release MPO and lead to oxidative stress. Through detecting MPO levels in gastric tissues, we found that the IG administration of 200 mg/kg ASP significantly augmented the content of MPO. However, the increased MPO content was reduced by the IG pre‐administration with 150 μg/kg JK‐1 (Fig. 5A). On the other hand, we further examined the levels of endogenous antioxidative defence system, reduced glutathione GSH, and found that the exposure to ASP markedly decreased the content of GSH in the mouse gastric mucosa, which was suppressed by the pre‐treatment with JK‐1 (Fig. 5B). The results suggest that the application of ASP breaks down the endogenous redox balance and causes the excessive oxidation, and these deleterious effects of ASP can be abrogated by the pre‐treatment with JK‐1.

### JK‐1 protected against ASP‐induced injury of endogenous gastric defence

**Figure 6:**
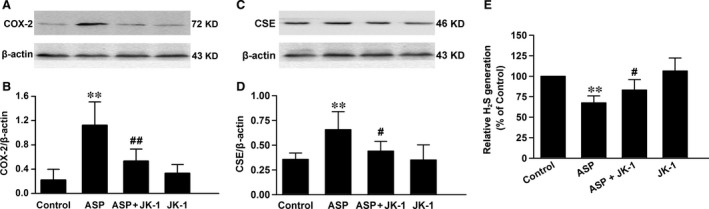
Effects of JK‐1 and ASP on endogenous gastric defence. The mice were fasted for 36 hrs with free access to drinking water and then were divided into four groups as *DRUG TREATMENTS AND EXPERIMENTAL DESIGN*: control group, ASP group, ASP+JK‐1 group and JK‐1 group. After the treatments, the mouse gastric mucosa was cut and split, and the protein samples were extracted for COX‐2 and CSE expression with Western blot assay. The endogenous H_2_S generation was measured in reaction bottles followed by methylene blue assay. Data were expressed as mean ± S.D. *n* = 4–6. **P <* *0.01, *versus* control group, #*P *<* *0.05, ##*P *<* *0.01 *versus* ASP alone group.

COX‐2 is an important endogenous protective enzyme in gastric mucosa *via* producing PGE_2_ under stress 20. We then performed experiments to observe its expression. As presented in Figure 6A and B, the IG administration of 200 mg/kg ASP markedly up‐regulated COX‐2 protein expression in the mouse gastric mucosa. However, the effects of ASP were significantly blocked by the preconditioning with 150 μg/kg JK‐1.

In addition, the above data of Figure 3F indicated that the exposure to ASP impaired the ability of gastric mucosa to generate H_2_S, another important gastric mucosal defence molecule 21. We then investigated the expression of CSE, a H_2_S synthetase, in ASP‐induced gastric injury. Through Western blot analysis, we found that the IG administration of 200 mg/kg ASP significantly up‐regulated CSE expression (Fig. 6C and D), but down‐regulated H_2_S generation (Fig. 6E) in the mouse gastric mucosa. However, both the overexpression of CSE and the down‐regulation of H_2_S generation were prevented by the pre‐treatment with JK‐1, indicating a pre‐administration of JK‐1 may prevent ASP‐induced H_2_S signal dysfunction.

### JK‐1 decreased HClO‐elicited cellular injury in GES‐1 cells

**Figure 7:**
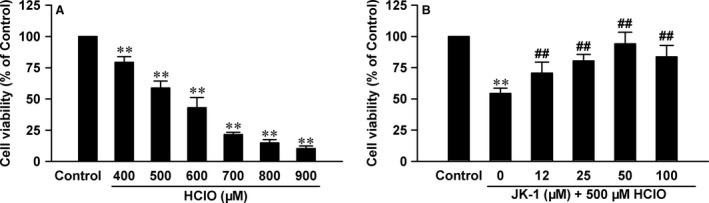
Effects of JK‐1 on HClO‐induced cellular injury in GES‐1 cells. (**A**) The cells were exposed to increasing concentrations of HClO ranging from 400 to 900 μM for 24 hrs. (**B**) Before the exposure to 500 μM HClO for 24 hrs, the cells were pre‐treated with various concentrations of JK‐1 for 1 hr. At the end of treatments, cell viability was measured by CCK‐8 assay. Data were shown as mean ± S.D. *n* = 5. **P <* *0.01 *versus* control group, ##*P *<* *0.01 *versus* HClO alone group.

As induction of MPO in activated neutrophils can lead to HClO generation, we performed experiment to directly investigate the effects of exogenous HClO on cultured human GES‐1 cells. The data showed that at the concentrations ranging from 400 to 900 μM, HClO dose‐dependently reduced cell viability (Fig. 7A), indicating the increased ROS levels from activated neutrophils *in vivo* were harmful to GES‐1 cells. We then observed the influence of JK‐1 on HClO‐induced cell injury and found that at the concentrations ranging from 12 to 50 μM, JK‐1 significantly decreased the destructive effects induced by the exogenous HClO (Fig. 7B).

### JK‐1 suppressed HClO‐induced apoptosis and mitochondrial injury in GES‐1 cells

**Figure 8:**
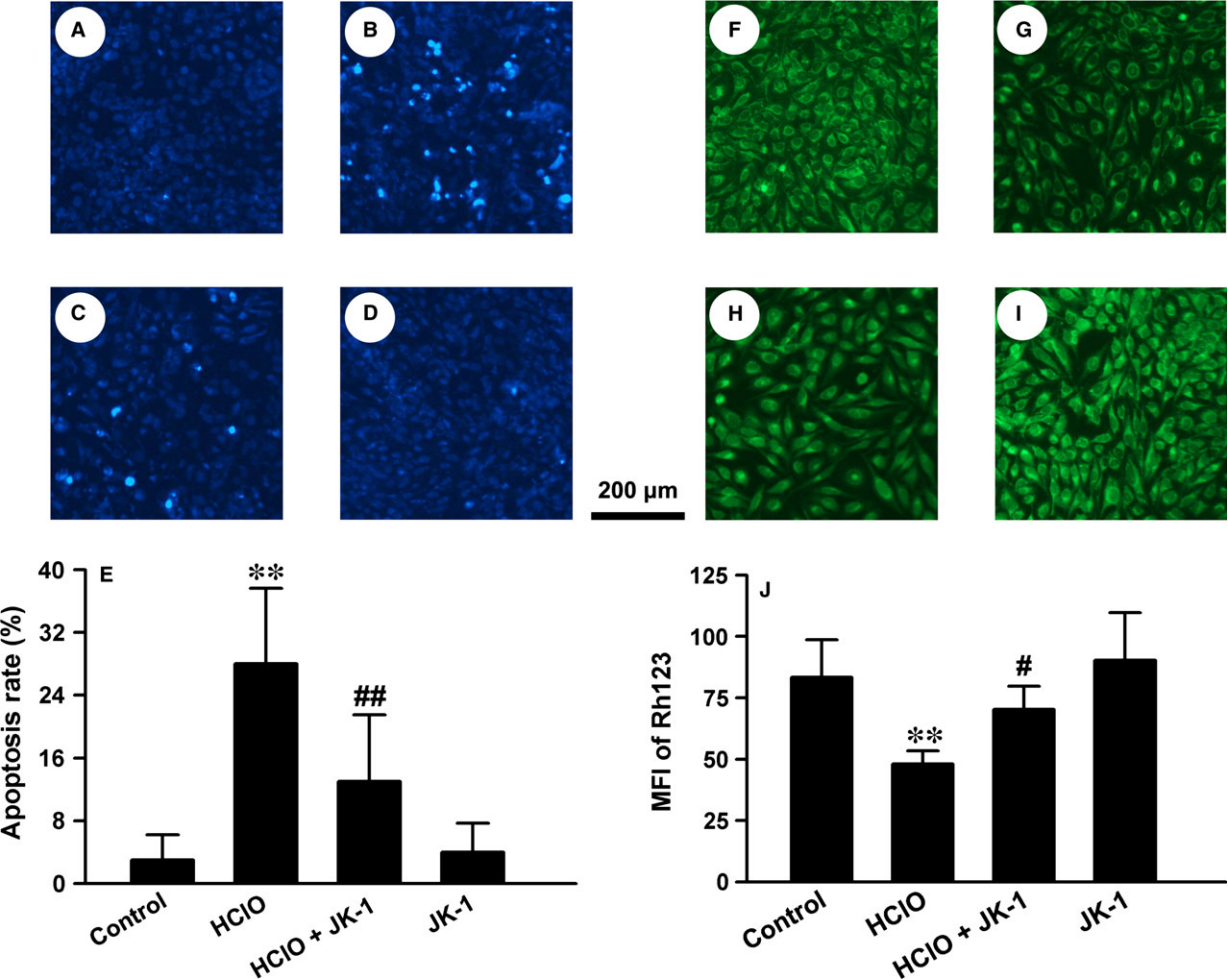
Effects of JK‐1 on HClO‐induced cellular apoptosis and MMP depolarization in GES‐1 cells. Cellular apoptosis and MMP were observed with Hoechst 33258 nuclear staining and Rh123 mitochondrial staining followed by photofluorography, respectively. Random fluorescent micrographs from the control cells (**A** and **F**), the cells treated with 500 μM HClO for 24 hrs (**B** and **G**), the cells pre‐treated with 50 μM JK for 1 hr before the exposure to HClO (**C** and **H**) and the cells treated with 50 μM JK for 1 hr followed by 24 hrs culture (**D** and **I**). The cellular apoptosis rate (**E**) and the mean fluorescence intensity (MFI) of Rh123 (**J**) were analysed with ImageJ software. Data were presented as mean ± S.D. **P <* *0.01 *versus* control group, #*P *<* *0.05, ##*P *< 0.01 *versus* HClO alone group.

Cellular apoptosis is one of the most important reasons for the decreased cell viability induced by various stimuli. As JK‐1 blunted HClO‐induced decrease in cell viability, we wanted to further clarify whether the cytoprotective effects of JK‐1 were associated with antiapoptosis. Therefore, Hoechst 33258 nuclear staining followed by photofluorography was performed to visualize cellular apoptosis. The data in Figure 8 showed that the nuclei of control cells exhibited weak and uniform fluorescence (A), so did those of JK‐1‐treated cells (D). However, when the cells were exposed to 500 μM HClO for 24 hrs, the number of apoptotic cells markedly increased (B and E) comparing with that of control cells. Importantly, HClO‐induced cell apoptosis was prevented by the pre‐treatment with 50 μM JK‐1 for 1 hr (C and E).

Mitochondrial dysfunction is a vital cause of cellular apoptosis and usually manifests depolarization of MMP. We used a mitochondrial fluorescent dye, Rh123, to measure MMP level. The results showed that when the cells were exposed to 500 μM HClO for 24 hrs, the MMP level of cells was obviously suppressed (G) comparing with that of control cells (F). Prior to the treatment with HClO, the cells were preconditioned with 50 μM JK‐1 for 1 hr. The results showed that the HClO‐triggered MMP depolarization was statistically attenuated by the pre‐treatment with JK‐1 (H), while it alone did not alter MMP levels (I). These data indicate that the improvement of MMP may underlie JK‐1′s antiapoptotic effects.

## Discussion

In the current work, we identified a pH‐controlled H_2_S donor JK‐1 and validated its applications in H_2_S‐relevant biological studies. We observed its gastric protection in ASP‐exposed mice and HClO‐stimulated GES‐1 cells. We demonstrated the protection effects were associated with the restoration of aberrant inflammatory and oxidative status, and the reconstruction of endogenous gastric defence. Our results should provide an insightful support for the treatment of adverse effects of NSAIDs with this kind of H_2_S donors in the future.

The acute gastric lesion induced by NSAIDs, including ASP, is a significant adverse side‐effect, which has limited their clinical usage. To solve this problem, a series of methods have been taken in recent years. Notably, some researchers have redesigned existing NSAIDs through conjugation with H_2_S and/or NO releasing agents 5, 13, 22, 23, 24. H_2_S and NO are endogenous gaseous signalling molecules with potent cytoprotective effects. Their beneficial effects are mainly associated with relaxation of blood vessels, and inhibition of oxidative injury and excessive inflammation 10, 11. Therefore, they have been used to reduce NSAIDs‐induced gastric 25. In this study, we administered intragastrically ASP to mice to imitate oral administration of NSAIDs in human. Our results indicated that the exposure of empty stomachs to ASP caused obvious festering and bleeding in mouse gastric mucosa. To examine the effects of JK‐1, a unique pH‐controlled H_2_S donor, on ASP‐induced acute gastric injury, the mice were pre‐administered intragastrically JK‐1 before the administration of ASP. The results showed that the treatment with JK‐1 significantly alleviated the ASP‐induced gastric injury, similar to those H_2_S‐releasing NSAIDs‐mediated gastric protection 5, 22. However, our current experimental design is different from those H_2_S‐releasing NSAIDs in the following aspects: (*i*) we do not need to change chemical structures of NSAIDs. Therefore, this process is relatively simple, without producing new drugs, altering pharmaceutical activity simultaneously or concerning further new drug test of various stages; (*ii*) comparing with currently known H_2_S‐releasing NSAIDs, H_2_S release from JK‐1 is controlled by acidic pH. Therefore, this type of donors may find many interesting applications as many pathological conditions cause acidic environments, such as ischaemic tissues 18. In addition, our results indicated that JK‐1 increased the solution's pH *via* consumption of proton. This may be significant for the therapy of gastric injury by means of antiacid, like proton pump inhibitors 20. Of course, we speculate that the effects of JK‐1, at a concentration of micromole, on the acidity in human stomach may be very limited because of the low pH value and should not be a main mechanism underlying the gastric protection.

Although NSAIDs are anti‐inflammatory drugs, they can still cause inflammatory response in gastric mucosa, evidenced by infiltration of leucocytes and release of pro‐inflammatory factors 6. It has been accepted that the inhibition of endogenous H_2_S generation is one of the crucial reasons for NSAIDs‐induced gastric inflammation 8, 9. In the present study, we found that besides the festering and bleeding of gastric mucosa, there were a large number of leucocytes in the submucosa of the mice exposed to ASP. However, in JK‐1‐treated mice the number of infiltrated leucocytes was distinctly reduced, while JK‐1 alone did not markedly affect the infiltration of inflammatory cells. Furthermore, we measured the content of pro‐inflammatory factors in gastric tissues and found that the administration of ASP significantly enhanced IL‐6 and TNF‐α release, which was attenuated by the preconditioning with JK‐1. These results suggested that in NSAIDs‐stimulated stomachs, H_2_S can exert anti‐inflammatory effects, which is also supported by a previous report 26. To our knowledge, the impacts of H_2_S on inflammation appear to be inconsistent. For example, in nervous system, H_2_S can also blunt inflammation response induced by lipopolysaccharide 27. However, in caecal ligation and puncture‐induced sepsis in mice, H_2_S can elicit inflammation in a NF‐κB‐dependent manner 28, 29. These conflicting studies, we surmise, may be related to various factors, such as special tissues, stimulating factors, as well as H_2_S's amount used or release rate.

In this study, we also found that the intragastric administration of ASP clearly triggered oxidative stress, evidenced by MPO release and GSH depletion. In fact, these findings are consistent with the above results on the ASP exposure‐induced leucocyte infiltration. As we know, once activated, leucocytes, especially neutrophils and monocytes, will release MPO and elastase and consequently damage epithelial tissues 30. Importantly, MPO can react with hydrogen peroxide (H_2_O_2_) to form a complex, which will oxidize a large variety of substances. Among the substances, chloride is an important candidate, which can be oxidized to hypochlorous acid (HClO). Researches have shown that HClO is a powerful oxidant that can damage adjacent tissues and cause inflammation 31, 32. Notably, in the present study, we used HClO to treat human GES‐1 cells and found that *in vitro* incubation of HClO reduced cellular viability, damaged mitochondrial function and caused apoptotic death. Furthermore, these lesions were markedly abated by the pre‐treatment with JK‐1. On the other hand, as a potent endogenous antioxidant, GSH can scavenge H_2_O_2_ and HClO and thus mitigate inflammatory injury 32, 33. Many reports and ours all indicated that the exposure to ASP could decrease the levels of GSH in stomachs 32, 33. Interestingly, our results further demonstrated that the ASP‐induced MPO release and GSH depletion were both partially reversed by the pre‐administration of JK‐1, indicating the antioxidative effects of H_2_S. This finding is also supported by other forms of H_2_S donors‐mediated antioxidation 18, 34, 35, 36.

Last but not the least, we investigated another endogenous gastric mucosal defence, COX, which is a rate‐limiting enzyme and can catalyse arachidonic acid to produce PGs. Different from the constitutive expression of COX‐1, the expression of COX‐2 is usually induced under stress condition. In this study, we presented that the exposure to ASP markedly up‐regulated COX‐2. The mechanisms may be associated with the inhibition of COX‐2 activity and PGE_2_ generation induced by ASP, which triggers an endogenous adaptive protection in gastric mucosa 5. And the induction of COX‐2 may partially relieve the PGE_2_ defect and thereby contribute to the repair of gastric mucosa. Importantly, we found that ASP‐induced COX‐2 up‐regulation was significantly attenuated by the pre‐administration of JK‐1. We think this may indicate that the lesions induced by ASP are alleviated, because the elevation of COX‐2 is a specific response to the injury induced by NSAIDs including ASP. In fact, Liu *et al*. also found that H_2_S‐releasing ASP had weaker effects on the COX‐2 induction than ASP alone 5. In addition, the supplement of PGE_2_ could also reduce ASP‐induced COX‐2 expression 37. These studies, alongside our findings, support that the inhibition of COX‐2 induction is an important aspect of H_2_S's gastric protection. The endogenously produced H_2_S *per se* is also a gastric mucosal defence. For example, it can induce HCO_3_^‐^ secretion involved in gastric mucosal barrier 38, improvement of microvascular blood flow, and balance of oxidation and inflammation status 10. Our findings showed that under ASP exposure, the ability of damaged gastric mucosa to produce H_2_S begun to weaken; however, the expression of CSE was increased. We think that the down‐regulated ability to generate H_2_S may be one of the pivotal reasons for the consequent ASP‐induced injury, and the up‐regulation of CSE may be similar to COX‐2 induction, that is an adaptive response to produce enough protective H_2_S. Of note, the further study showed that the pre‐treatment with JK‐1 reversed the ASP‐induced changes both in CSE expression and H_2_S generation, which means that the exogenous applied JK‐1 can correct the aberrant endogenous CSE/H_2_S signal.

In conclusion, the current study showed a pH‐controlled H_2_S donor, JK‐1, could release H_2_S under acidic pH value through H^+^ consumption. Importantly, it could prevent ASP‐induced gastric lesions *via* reducing inflammation and oxidation, as well as reconstructing endogenous gastric defence. *In vitro* experiments suggested that JK‐1 could improve mitochondrial function and inhibit apoptotic death. These results may provide a basal support for the treatment of NSAID injury with pH‐controlled H_2_S donors in the future.

## Conflict of interest

The authors have nothing to disclose.

## Author contribution

Chun‐tao Yang, Ming Xian and Hui Zhang participated in the research design. Zhen‐zhen Lai, Ze‐hang Zheng, Jian‐ming Kang, Rui‐yu Wang, Kun Shi, Fu‐hui Meng, Xiang Li and Li Chen performed the experiment and analysed the data. . Chun‐tao Yang, Ming Xian and Hui Zhang wrote or contributed to the writing of the manuscript.

## References

[1] Wallace JL . Prostaglandins, NSAIDs, and gastric mucosal protection: why doesn't the stomach digest itself? Physiol Rev. 2008; 88: 1547–65. 18923189 10.1152/physrev.00004.2008

[2] Scheiman JM . Prevention of NSAID‐Induced Ulcers. Curr Treat Options Gastroenterol. 2008; 11: 125–34. 18321440 10.1007/s11938-008-0025-7

[3] Wallace JL . NSAID gastropathy and enteropathy: distinct pathogenesis likely necessitates distinct prevention strategies. Br J Pharmacol. 2012; 165: 67–74. 21627632 10.1111/j.1476-5381.2011.01509.x PMC3252967

[4] McCarthy DM . GI bleeding: problems that persist. Gastrointest Endosc. 2009; 70: 225–8. 19631801 10.1016/j.gie.2008.12.247

[5] Liu L , Cui J , Song CJ , *et al* H_2_S‐releasing aspirin protects against aspirin‐induced gastric injury *via* reducing oxidative stress. PLoS One. 2012; 7: e46301. 23029468 10.1371/journal.pone.0046301 PMC3460860

[6] Souza MH , Lemos HP , Oliveira RB , *et al* Gastric damage and granulocyte infiltration induced by indomethacin in tumour necrosis factor receptor 1 (TNF‐R1) or inducible nitric oxide synthase (iNOS) deficient mice. Gut. 2004; 53: 791–6. 15138204 10.1136/gut.2002.012930 PMC1774069

[7] Konturek PC , Celinski K , Slomka M , *et al* Melatonin and its precursor L‐tryptophan prevent acute gastric mucosal damage induced by aspirin in humans. J Physiol Pharmacol. 2008; 59: 67–75. 18812629

[8] Fiorucci S , Antonelli E , Distrutti E , *et al* Inhibition of hydrogen sulfide generation contributes to gastric injury caused by anti‐inflammatory nonsteroidal drugs. Gastroenterology. 2005; 129: 1210–24. 16230075 10.1053/j.gastro.2005.07.060

[9] Zayachkivska O , Bula N , Khyrivska D , *et al* Exposure to non‐steroid anti‐inflammatory drugs (NSAIDs) and suppressing hydrogen sulfide synthesis leads to altered structure and impaired function of the oesophagus and oesophagogastric junction. Inflammopharmacology. 2015; 23: 91–9. 25711289 10.1007/s10787-015-0230-7

[10] Wang R . Physiological implications of hydrogen sulfide: a whiff exploration that blossomed. Physiol Rev. 2012; 92: 791–896. 22535897 10.1152/physrev.00017.2011

[11] Pacher P , Beckman JS , Liaudet L . Nitric oxide and peroxynitrite in health and disease. Physiol Rev. 2007; 87: 315–424. 17237348 10.1152/physrev.00029.2006 PMC2248324

[12] Sparatore A , Perrino E , Tazzari V , *et al* Pharmacological profile of a novel H_2_S‐releasing aspirin. Free Radic Biol Med. 2009; 46: 586–92. 19100325 10.1016/j.freeradbiomed.2008.11.013

[13] Amoruso A , Fresu LG , Dalli J , *et al* Characterization of the anti‐inflammatory properties of NCX 429, a dual‐acting compound releasing nitric oxide and naproxen. Life Sci. 2015; 126: 28–36. 25711428 10.1016/j.lfs.2015.01.025

[14] Geusens P . Naproxcinod, a new cyclooxygenase‐inhibiting nitric oxide donator (CINOD). Expert Opin Biol Ther. 2009; 9: 649–57. 19392579 10.1517/14712590902926071

[15] Kashfi K , Chattopadhyay M , Kodela R . NOSH‐sulindac (AVT‐18A) is a novel nitric oxide‐ and hydrogen sulfide‐releasing hybrid that is gastrointestinal safe and has potent anti‐inflammatory, analgesic, antipyretic, anti‐platelet, and anti‐cancer properties. Redox Biol. 2015; 6: 287–96. 26298203 10.1016/j.redox.2015.08.012 PMC4556776

[16] Kodela R , Chattopadhyay M , Velazquez‐Martinez CA , *et al* NOSH‐aspirin (NBS‐1120), a novel nitric oxide‐ and hydrogen sulfide‐releasing hybrid has enhanced chemo‐preventive properties compared to aspirin, is gastrointestinal safe with all the classic therapeutic indications. Biochem Pharmacol. 2015; 98: 564–72. 26394025 10.1016/j.bcp.2015.09.014 PMC4656078

[17] Li L , Whiteman M , Guan YY , *et al* Characterization of a novel, water‐soluble hydrogen sulfide‐releasing molecule (GYY4137): new insights into the biology of hydrogen sulfide. Circulation. 2008; 117: 2351–60. 18443240 10.1161/CIRCULATIONAHA.107.753467

[18] Kang J , Li Z , Organ CL , *et al* pH‐Controlled Hydrogen Sulfide Release for Myocardial Ischemia‐Reperfusion Injury. J Am Chem Soc. 2016; 138: 6336–9. 27172143 10.1021/jacs.6b01373

[19] Zhang H , Zhuang XD , Meng FH , *et al* Calcitriol prevents peripheral RSC96 Schwann neural cells from high glucose & methylglyoxal‐induced injury through restoration of CBS/H_2_S expression. Neurochem Int. 2016; 92: 49–57. 26707812 10.1016/j.neuint.2015.12.005

[20] Laine L , Takeuchi K , Tarnawski A . Gastric mucosal defense and cytoprotection: bench to bedside. Gastroenterology. 2008; 135: 41–60. 18549814 10.1053/j.gastro.2008.05.030

[21] Farrugia G , Szurszewski JH . Carbon monoxide, hydrogen sulfide, and nitric oxide as signaling molecules in the gastrointestinal tract. Gastroenterology. 2014; 147: 303–13. 24798417 10.1053/j.gastro.2014.04.041 PMC4106980

[22] Chattopadhyay M , Kodela R , Duvalsaint PL , *et al* Gastrointestinal safety, chemotherapeutic potential, and classic pharmacological profile of NOSH‐naproxen (AVT‐219) a dual NO‐ and H_2_S‐releasing hybrid. Pharmacol Res Perspect. 2016; 4: e00224. 27069635 10.1002/prp2.224 PMC4804313

[23] Wallace JL . Hydrogen sulfide‐releasing anti‐inflammatory drugs. Trends Pharmacol Sci. 2007; 28: 501–5. 17884186 10.1016/j.tips.2007.09.003

[24] Wallace JL , Caliendo G , Santagada V , *et al* Markedly reduced toxicity of a hydrogen sulphide‐releasing derivative of naproxen (ATB‐346). Br J Pharmacol. 2010; 159: 1236–46. 20128814 10.1111/j.1476-5381.2009.00611.x PMC2848928

[25] Wallace JL . Physiological and pathophysiological roles of hydrogen sulfide in the gastrointestinal tract. Antioxid Redox Signal. 2010; 12: 1125–33. 19769457 10.1089/ars.2009.2900

[26] Zanardo RC , Brancaleone V , Distrutti E , *et al* Hydrogen sulfide is an endogenous modulator of leukocyte‐mediated inflammation. Faseb J. 2006; 20: 2118–20. 16912151 10.1096/fj.06-6270fje

[27] Hu LF , Wong PT , Moore PK , *et al* Hydrogen sulfide attenuates lipopolysaccharide‐induced inflammation by inhibition of p38 mitogen‐activated protein kinase in microglia. J Neurochem. 2007; 100: 1121–8. 17212697 10.1111/j.1471-4159.2006.04283.x

[28] Zhang H , Zhi L , Moochhala S , *et al* Hydrogen sulfide acts as an inflammatory mediator in cecal ligation and puncture‐induced sepsis in mice by upregulating the production of cytokines and chemokines *via* NF‐kappaB. Am J Physiol Lung Cell Mol Physiol. 2007; 292: L960–71. 17209138 10.1152/ajplung.00388.2006

[29] Li L , Bhatia M , Zhu YZ , *et al* Hydrogen sulfide is a novel mediator of lipopolysaccharide‐induced inflammation in the mouse. FASEB J. 2005; 19: 1196–8. 15863703 10.1096/fj.04-3583fje

[30] Saffarzadeh M , Juenemann C , Queisser MA , *et al* Neutrophil extracellular traps directly induce epithelial and endothelial cell death: a predominant role of histones. PLoS One. 2012; 7: e32366. 22389696 10.1371/journal.pone.0032366 PMC3289648

[31] Klebanoff SJ . Myeloperoxidase. Proc Assoc Am Physicians. 1999; 111: 383–9. 10519157 10.1111/paa.1999.111.5.383

[32] Pullar JM , Vissers MC , Winterbourn CC . Living with a killer: the effects of hypochlorous acid on mammalian cells. IUBMB Life. 2000; 50: 259–66. 11327319 10.1080/713803731

[33] Aoyama K , Nakaki T . Inhibition of GTRAP3‐18 may increase neuroprotective glutathione (GSH) synthesis. Int J Mol Sci. 2012; 13: 12017–35. 23109897 10.3390/ijms130912017 PMC3472789

[34] Xie L , Feng H , Li S , *et al* SIRT3 Mediates the Antioxidant Effect of Hydrogen Sulfide in Endothelial Cells. Antioxid Redox Signal. 2016; 24: 329–43. 26422756 10.1089/ars.2015.6331 PMC4761821

[35] Yang C , Yang Z , Zhang M , *et al* Hydrogen Sulfide Protects against Chemical Hypoxia‐Induced Cytotoxicity and Inflammation in HaCaT Cells through Inhibition of ROS/NF‐kappaB/COX‐2 Pathway. PLoS One. 2011; 6: e21971. 21779360 10.1371/journal.pone.0021971 PMC3136491

[36] Yang G , Zhao K , Ju Y , *et al* Hydrogen sulfide protects against cellular senescence *via* S‐sulfhydration of Keap1 and activation of Nrf2. Antioxid Redox Signal. 2013; 18: 1906–19. 23176571 10.1089/ars.2012.4645

[37] Davies NM , Sharkey KA , Asfaha S , *et al* Aspirin causes rapid up‐regulation of cyclo‐oxygenase‐2 expression in the stomach of rats. Aliment Pharmacol Ther. 1997; 11: 1101–8. 9663836 10.1046/j.1365-2036.1997.00247.x

[38] Takeuchi K , Ise F , Takahashi K , *et al* H_2_S‐induced HCO_3_ ^‐^ secretion in the rat stomach–involvement of nitric oxide, prostaglandins, and capsaicin‐sensitive sensory neurons. Nitric Oxide. 2015; 46: 157–64. 25460323 10.1016/j.niox.2014.11.001

